# Protein kinase C signaling and cell cycle regulation

**DOI:** 10.3389/fimmu.2012.00423

**Published:** 2013-01-17

**Authors:** Adrian R. Black, Jennifer D. Black

**Affiliations:** Eppley Institute for Research in Cancer and Allied Diseases, University of Nebraska Medical CenterOmaha, NE, USA

**Keywords:** protein kinase C, signal transduction, T cell activation, cell cycle, cyclin, cyclin-dependent kinase, cyclin-dependent kinase inhibitor

## Abstract

A link between T cell proliferation and the protein kinase C (PKC) family of serine/threonine kinases has been recognized for about 30 years. However, despite the wealth of information on PKC-mediated control of, T cell activation, understanding of the effects of PKCs on the cell cycle machinery in this cell type remains limited. Studies in other systems have revealed important cell cycle-specific effects of PKC signaling that can either positively or negatively impact proliferation. The outcome of PKC activation is highly context-dependent, with the precise cell cycle target(s) and overall effects determined by the specific isozyme involved, the timing of PKC activation, the cell type, and the signaling environment. Although PKCs can regulate all stages of the cell cycle, they appear to predominantly affect G0/G1 and G2. PKCs can modulate multiple cell cycle regulatory molecules, including cyclins, cyclin-dependent kinases (cdks), cdk inhibitors and cdc25 phosphatases; however, evidence points to Cip/Kip cdk inhibitors and D-type cyclins as key mediators of PKC-regulated cell cycle-specific effects. Several PKC isozymes can target Cip/Kip proteins to control G0/G1 → S and/or G2 → M transit, while effects on D-type cyclins regulate entry into and progression through G1. Analysis of PKC signaling in T cells has largely focused on its roles in T cell activation; thus, observed cell cycle effects are mainly positive. A prominent role is emerging for PKCθ, with non-redundant functions of other isozymes also described. Additional evidence points to PKCδ as a negative regulator of the cell cycle in these cells. As in other cell types, context-dependent effects of individual isozymes have been noted in T cells, and Cip/Kip cdk inhibitors and D-type cyclins appear to be major PKC targets. Future studies are anticipated to take advantage of the similarities between these various systems to enhance understanding of PKC-mediated cell cycle regulation in T cells.

An association between protein kinase C (PKC) signaling and T cell proliferation has been recognized for almost three decades. Over 30 years ago, it was determined that a combination of phorbol esters and elevated intracellular calcium potently induces proliferation of cells of the T cell lineage (e.g., Whitfield et al., 1973; Lyall et al., 1980; Sutherland et al., 1981). Shortly thereafter, it was determined that PKC, then recognized as a calcium-dependent enzyme, represented the major cellular receptor for phorbol esters (Castagna et al., 1982) and it was not long before the connection between these phenomena was made (Isakov et al., 1986; Isakov and Altman, 1987). Since then, a central role for PKC in T cell receptor (TCR) signaling has been firmly established. Despite the long association of PKC with T cell proliferation, details on how PKC signaling interacts with the cell cycle machinery in this cell type are only beginning to emerge. In this regard, our knowledge in T cells lags behind that in other cell types, including other hematopoietic lineages. In this review, we outline our current understanding of the proliferative effects of PKC signaling in T cells within the context of the broader knowledge that has been gained in other systems.

## THE PROTEIN KINASE C FAMILY

Protein kinase C represents a family of serine/threonine kinases that belong to the AGC (cAMP-dependent, cGMP-dependent, and protein kinase C) superfamily of protein kinases (Nieto, 2007; Matsuoka et al., 2009; Black, 2010; Rosse et al., 2010; Ryu et al., 2010). PKC isozymes are lipid-dependent kinases (requiring phosphatidylserine binding for activity) and are grouped into three subfamilies based on their structure and requirement for additional co-factors and calcium. Physiological activation of classical PKCs (PKCα, PKCβI and PKCβII, which are splice variants of the *prkcb* gene, and PKCγ) is induced by the lipid second messenger diacylglycerol (DAG) and calcium, while activation of the novel PKCs (PKCδ, PKCε, PKCθ, and PKCη) requires only DAG. In contrast, the atypical PKCs (PKCζ and PKCι/λ) are not dependent on lipid second messengers or calcium for activity. Instead, their function is regulated by protein–protein interactions mediated by a PB1 domain as well as a carboxyl-terminal PDZ ligand motif. Engagement of growth factor or cytokine receptors leads to activation of phospholipase C (PLC) β or PLCγ, which cleave phosphatidylinositol 4,5-bisphosphate to generate DAG and the soluble second messenger inositol trisphosphate (which induces release of calcium from intracellular stores). The production of DAG recruits classical and novel PKCs to the plasma membrane, where they undergo a conformational change resulting in full activation. Unlike other AGC kinases, such as Akt, activation of PKCs does not require acute phosphorylation of the enzyme: phosphorylations necessary for catalytic competence occur shortly after synthesis and the enzyme is constitutively phosphorylated at these sites (Matsuoka et al., 2009; Rosse et al., 2010). As a result, changes in phosphorylation do not provide an indication of PKC activity; rather signaling-induced translocation of the enzyme to the membrane/particulate fraction represents the most reliable means of monitoring kinase activation. Reversal of signaling can occur by metabolism of DAG by DAG kinase and release of PKCs from the membrane, as well as by agonist-induced enzyme degradation or removal of priming phosphorylation with subsequent rapid degradation (Leontieva and Black, 2004; Newton, 2010). In addition to activation by growth factor signaling, classical and novel PKCs can be stimulated by a number of pharmacological agents that mimic the effects of DAG, such as phorbol esters and macrocyclic lactone bryostatins. However, in contrast to DAG, these agonists, which include phorbol 12-myristate 13-acetate [PMA; also known as 12-*O*-tetradecanoylphorbol-13-acetate (TPA)], phorbol 12,13-dibutyrate (PDBu), and bryostatin 1, are not rapidly metabolized and thus give a more sustained PKC activation.

Despite limitations related to their lack of specificity for individual PKC isozymes, their ability to promote PKC downregulation, and the existence of additional targets for these agents (Griner and Kazanietz, 2007), use of pharmacological agonists and membrane permeant DAG analogs has provided significant insight into the downstream effects of PKC activation. However, a complete understanding of PKC signaling will require defining the specific function(s) of individual PKC isozymes, and progress toward this goal has proved technically difficult. Understanding of the functions of atypical PKCs, PKCζ, and PKCι, lags behind that of other members of the PKC family, perhaps largely due to their insensitivity to pharmacological activators (e.g., phorbol esters and bryostatins) and synthetic DAGs. In the absence of isozyme-specific pharmacological PKC agonists and inhibitors, early studies relied on overexpression strategies to decipher the roles of individual isozymes, which can result in non-physiological levels of expression, activity, and regulation. RNA interference technology and genetically altered mice are helping to circumvent these problems, but are not without drawbacks of their own. Potential limitations include the need for a high level of silencing to sufficiently deplete enzyme activity (e.g.,>80%, Cameron et al., 2008; M. A. Pysz, A. R. Black, and J. D. Black, unpublished results), and the fact that knockdown of one PKC isozyme can affect accumulation of other members of the family (M. A. Pysz, A. R. Black, and J. D. Black, unpublished data). Overlapping roles of different isozymes means that multiple crosses of transgenic mice may be needed to observe phenotypes.

An additional source of confusion regarding the functions of individual PKC isozymes is the fact that many so-called PKC inhibitors are of questionable specificity (Griner and Kazanietz, 2007; Soltoff, 2007). As an example of particular relevance to T cell activation, special caution is needed when considering studies that have used rottlerin to infer effects of signaling from PKCθ. While this agent was originally considered to be a specific inhibitor of novel PKCs, recent studies have demonstrated that it does not inhibit PKCδ (Soltoff, 2007). In keeping with this finding, the IC_50_ for PKCθ inhibition by rottlerin in the presence of 100 μM ATP is>300 μM (Villalba et al., 1999), a concentration far in excess of that used in studies on its cellular effects. In contrast, rottlerin is a potent inhibitor of other kinases such as PRAK and MAPKAP-K2 (Soltoff, 2007); thus, any effects of this inhibitor cannot be ascribed to direct inhibition of PKCθ.

Despite these limitations, our knowledge of the roles of individual PKCs is emerging. Of note, in addition to the proliferative/cell cycle effects which are the subject of this review, PKC isozymes have been found to regulate multiple cellular processes of direct relevance to T cell development and function, including differentiation, migration, survival, apoptosis, endocytosis, and secretion/exocytosis (Reyland, 2009; Rosse et al., 2010).

## THE MAMMALIAN CELL CYCLE

**FIGURE 1:**
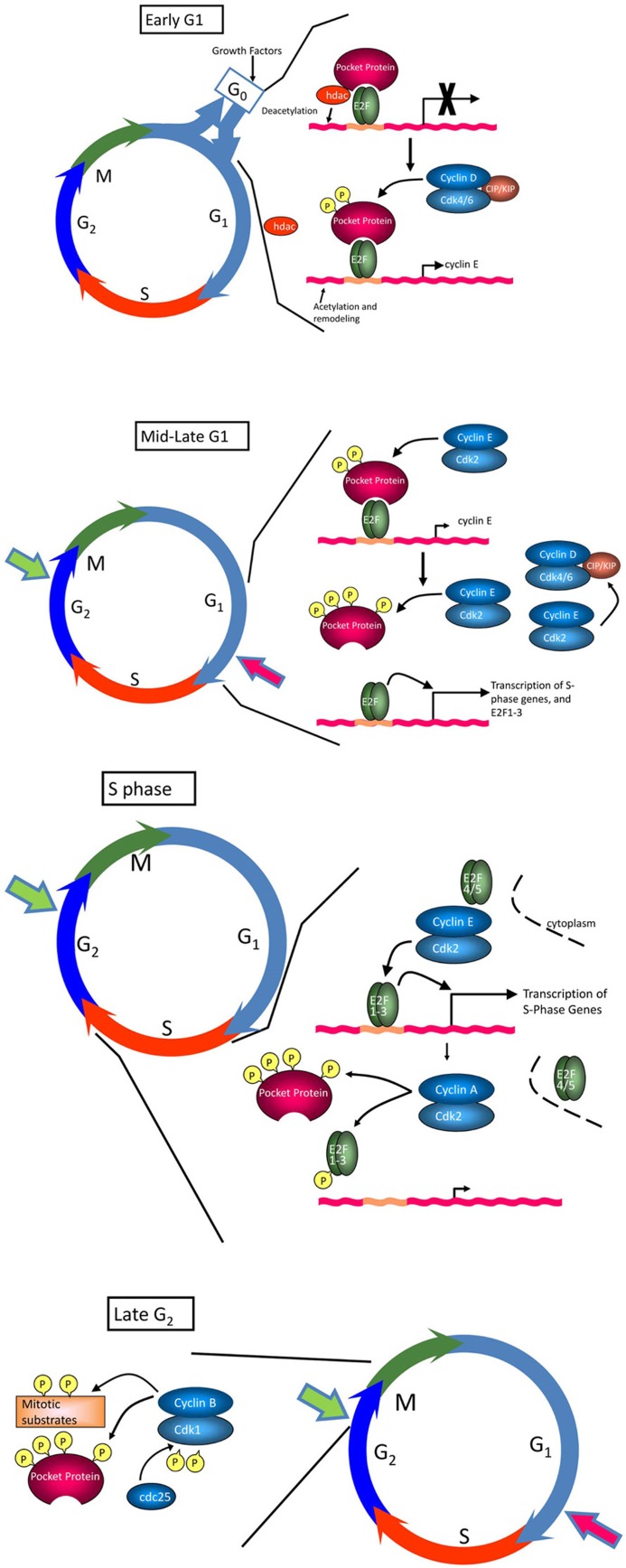
**The cell cycle**. The cell cycle consists of four phases, G1, S, G2, and M. In early G1, hypophosphorylated pRb binds the E2F transcription factor, and recruits histone deacetylase (HDAC) and other factors to actively repress transcription of E2F-regulated genes important for transition into S phase and DNA replication (e.g., PCNA, topoisomerase I, c-Myc, cyclin E, Cdc25c). Progression through early G1 is dependent on growth factors, which promote expression of D-type cyclins. Formation of cyclin D/cdk4 and cyclin D/cdk6 complexes, which is facilitated by Cip/Kip ckis, leads to phosphorylation of pRb at a subset of available sites and release of HDAC and other inhibitory factors, relieving repression of E2F and promoting upregulation of cyclin E. Cyclin E/cdk2 complexes, relieved from repression by Cip/Kip ckis by sequestration of these inhibitory molecules in cyclin D/cdk complexes, complete pocket protein phosphorylation in mid to late G1, enabling a wave of E2F-dependent transcriptional activity essential for S progression. Together, these events drive cells through the restriction point (large red arrow), which commits cells to the proliferative cycle. If conditions are not optimal to signal this transition, cells exit the cycle and enter G0 or quiescence, a reversible non-replicative state. Once cells enter S phase, cyclin E/cdk2 activity is inhibited by proteasomal degradation of cyclin E in the cytoplasm. Continued inactivation/hyperphosphorylation of pRb allows the transcription of cyclin A and cyclin B, required for subsequent phases of the cell cycle. Cyclin A/cdk2 complexes phosphorylate a number of proteins to facilitate S phase completion and transit into G2/M. Cyclin B is actively synthesized during G2 and associates with cdk1 to trigger mitosis. Cdk1 is maintained in an inactive state by the kinases, Wee1 and Myt1. As cells approach M phase, the phosphatase cdc25 is activated to remove inhibitory phosphates on Tyr14 and Thr15, driving the cells into mitosis. A checkpoint in late G2 (large green arrow) prevents cells from entering M phase if the genome is damaged. This DNA damage checkpoint ensures that cells do not initiate mitosis until they have repaired damaged DNA after replication.

Several excellent reviews have been written on the regulation of the cell cycle (Sherr and Roberts, 2004; Cobrinik, 2005; Malumbres and Barbacid, 2005; Du and Pogoriler, 2006; Satyanarayana and Kaldis, 2009) and only a brief description will be given here. The cell cycle has been classically divided into four phases, G1 (or Gap 1 in which cells prepare for DNA synthesis), S phase (in which DNA is synthesized), G2 (in which cells prepare for division) and mitosis (or M phase, in which sister chromatids are separated and the cell divides; **Figure 1**). Transit through the cell cycle is regulated by four major classes of cyclins whose expression is strictly controlled and limited to particular cell cycle phases. Cyclins are the regulatory subunits for cyclin-dependent kinases (cdks), whose activity is absolutely dependent on association with specific cyclin partners. Entry of quiescent cells into the cell cycle and transit through early G1 is regulated by D-type cyclins, which complex with cdk4 and cdk6 (Musgrove et al., 2011). There are three D-type cyclins, D1, D2, and D3, which are expressed to varying degrees in different tissues; cyclins D2 and D3 appear to be the major players in T cells. Transit through late G1 and progression into S phase is regulated by cyclin E complexed with cdk2 (Hwang and Clurman, 2005; Malumbres and Barbacid, 2005). S phase transit and early G2 are regulated by cyclin A/cdk2 and cyclin A/cdk1 complexes, whereas cyclin B, complexed with cdk1, regulates progression into M phase (Malumbres and Barbacid, 2005; Sanchez and Dynlacht, 2005). In addition to being regulated by cyclin binding, the activity of cdks is under the control of Cip/Kip and Ink4 cdk inhibitor proteins (ckis; Sherr and Roberts, 1995). Members of the Cip/Kip family, including p21^Cip1^, p27^Kip1^, and p57^Kip2^, have a dual activity in cell cycle regulation. They negatively regulate cell cycle progression by binding to cyclin/cdk2 and cyclin/cdk1 complexes and inhibiting their enzymatic activity. Conversely, these proteins can promote progression by enhancing the association of cyclin D with cdk4 and cdk6 without inhibiting the activity of these complexes (Sherr and Roberts, 1999). The Ink4 ckis, which include p15^Ink4b^, p16^Ink4a^, p18^Ink4c^, and p19^Ink4d^, block the activity of cdk4 and cdk6 by preventing their association with cyclin D. Cdk activity is also regulated by phosphorylation: positive phosphorylation is mediated by cdk activating kinase (CAK or cdk7/cyclin H; Fisher and Morgan, 1994), while negative phosphorylation involves the kinases Wee1 and Myt1. Removal of inhibitory phosphorylation, by e.g., Cdc25 phosphatases, is necessary for full cdk activity.

While there are multiple checkpoints that allow cells to undergo cell cycle arrest in response to various stresses, the most relevant to normal tissue homeostasis and differentiation is that which directs entry and exit from the cell cycle in G1 (Prasad et al., 1994; Liu et al., 2012). The expression of D-type cyclins is acutely regulated by mitogenic signals. As such, these proteins are the main sensors for the growth environment of the cell, and are intimately involved in regulation of the entry of quiescent cells into the cell cycle. Major targets for cyclin D/cdk complexes include the retinoblastoma protein (pRb) and related pocket proteins, p107 and p130 (Cobrinik, 2005). In the hypophosphorylated state, pocket proteins bind to E2F transcription factors on the promoters of growth-related genes, where they act as transcriptional repressors and actively block expression of genes necessary for DNA replication (Trimarchi and Lees, 2002; Du and Pogoriler, 2006; **Figure 1**). Phosphorylation of pocket proteins relieves this repression, allowing for transcription of E2F-dependent genes, one of which is cyclin E. Cyclin E/cdk2 then completes phosphorylation of pocket proteins, leading to their release from E2F and robust transcription of growth-related genes. At early stages of G1, cells require mitogenic signals to support cyclin expression and cdk activity; however, once sufficient levels of cyclin E have accumulated to maintain its own expression, cells have passed the so-called “restriction point” and are able to proceed through to the next cell cycle without further mitogenic input. In the face of loss of mitogenic signals prior to the restriction point or of negative growth signals, cell cycle progression is halted and cells eventually withdraw into G0 phase and quiescence (Grana et al., 1998; Classon and Dyson, 2001).

## PKC SIGNALING AND T CELL PROLIFERATION

### T CELL DEVELOPMENT AND TCR SIGNALING

T lymphocytes arise from bone marrow-derived CD34^+^ stem cells, which seed the thymus and undergo multistage differentiation to become mature circulating cells (for references, see Koch and Radtke, 2011). An early event in this process involves VDJ recombination of the TCR-β chain which then complexes with pre-Tα to form the pre-TCR. Signaling from the pre-TCR leads to proliferation of pre-T cells and rearrangement of the TCR-α chain, which combines with the β chain and CD3 to form the TCR. Further differentiation, accompanied by negative and positive selection, eventually leads to the development of mature naïve T cells, including CD4^+^ helper T (Th), CD8^+^ cytotoxic T (Tc), and regulatory T (Treg) cells. These naïve cells exit the thymus and remain dormant as they circulate through secondary lymphoid organs until activated by antigen. These organs, which include the spleen, lymph nodes, and Peyer’s patches, transiently house naïve T cells and are the first line of defense against pathogens that traverse the skin or the epithelial lining of the respiratory, gastrointestinal, and urogenital tracts.

Activation of T cells requires interaction of the TCR with major histocompatibility complex (MHC) bound antigen on antigen presenting cells (APCs), such as dendritic cells, macrophages, and B cells (for references, see Marsland and Kopf, 2008; Smith-Garvin et al., 2009; Fooksman et al., 2010; Dustin and Depoil, 2011). The interface between the T cell and APC is marked by the formation of a structure, termed the immune synapse or supramolecular activation cluster (SMAC), which serves to regulate T cell signaling. Productive activation of T cells requires two signals. The first signal is provided by the MHC-bound TCR, while the second signal is provided by co-stimulatory molecules such as CD28 (which binds to B7 proteins on the APC). Additionally, cytokines such as IL-12 and tumor necrosis factor alpha (TNF-α) can provide a third signal that regulates the response to T cell activation. A number of experimental manipulations can activate T cells in the absence of APC interaction; these include crosslinking of the TCR and CD28 with insoluble antibodies and combined treatment of cells with phorbol ester and calcium ionophore.

T cell receptor co-activation leads to the engagement of multiple downstream signaling pathways including those involving phosphatidylinositol 3-kinase (PI-3K), tyrosine kinases such as Lck, and PLCγ (for references, see Altman et al., 2000; Marsland and Kopf, 2008; Smith-Garvin et al., 2009; Fooksman et al., 2010; Dustin and Depoil, 2011). Activation of PLCγ results in production of DAG, which recruits PKCθ to the immune synapse where it interacts indirectly with CD28 through binding to Lck (Kong et al., 2011; Isakov and Altman, 2012). PKCθ then phosphorylates CARMA1, leading to the assembly of the CARMA1–BCL10–MALT1 (CBM) signalosome. PLCγ-generated inositol trisphosphate releases calcium from intracellular stores. The combined action of downstream TCR signaling eventually leads to activation of NF-κB, AP1, and nuclear factor of activated T cells (NFAT) transcription factors (Marsland and Kopf, 2008; Smith-Garvin et al., 2009; Fooksman et al., 2010; Dustin and Depoil, 2011). Together, these events promote functional activation of T cells which is marked by cell proliferation/clonal expansion and cytokine secretion. While the majority of the T cells that arise from activation are eventually cleared from the circulation, a small number develop into memory T cells which are primed for activation upon subsequent antigen exposure.

Antigen-induced proliferation is a key aspect of both T cell differentiation and clonal expansion (Koch and Radtke, 2011). Thus, mechanisms underlying regulation of the T cell cycle machinery are of critical importance to immune function. As the signaling pathways involved in T cell activation are being deciphered, increasing evidence is pointing to the importance of the PKC family in mediating proliferative responses in these cells. The following section outlines our current understanding of the role of individual PKC isozymes in regulating proliferation in T cells within the context of knowledge gained from other systems.

## PKCs AND THE CELL CYCLE

As our knowledge of the proliferative roles of the PKC family has developed, it has become increasingly apparent that the effects of these molecules are highly context-dependent. The fact that PKCs are activated by tumor promoting phorbol esters and are downstream of growth factor receptors initially led to the idea that they transduce positive mitogenic signals (Castagna et al., 1982; Kikkawa et al., 1983; Leach et al., 1983). Although a number of early studies supported this idea (Dicker and Rozengurt, 1978; Rozengurt, 1986; Takuwa et al., 1988), it soon became clear that PKCs can negatively and positively regulate cell cycle progression. Indeed, regulation of proliferation by the PKC enzyme system exhibits a high degree of complexity, with effects involving multiple cell cycle regulatory molecules, including cyclins, cdks, and ckis, and impacting various stages of the cell cycle (Black, 2010). Furthermore, individual isozymes can have opposing effects on cell cycle progression in different cell types and even within the same cell type, depending on the signaling environment. A single isozyme can target different cell cycle molecules in different cell types, can have opposite effects on a specific cell cycle target in different systems, and can modulate the same target to produce divergent cell cycle responses (for review, see Black, 2010). Thus, to gain a true understanding of the role of PKCs in regulation of proliferation in any given system, it is important to study the mechanisms by which individual isozymes affect specific cell cycle molecules in that system.

T lymphocytes express all members of the PKC family with the exception of PKCγ (Koretzky et al., 1989; Chen et al., 1994; Thuille et al., 2006). A role for PKC isozymes in cell cycle regulation in CD3^+^ T lymphocytes was suggested by the early recognition that phorbol esters, in conjunction with calcium ionophore, are potent mitogens for these cells (Altman et al., 1990). While studies have concentrated largely on the role of PKCθ in mediating signaling from the immune synapse, a role for other PKC isozymes is emerging. Notably, different isozymes can have pro-proliferative and/or anti-proliferative functions, arguing that, as in other cell types, PKC signaling can regulate entry into the cell cycle, transit through the various cell cycle phases, as well as cell cycle withdrawal in T cells. The following sections discuss current understanding of the growth regulatory functions of individual PKC family members, followed by a summary of the limited information available on cell cycle-specific effects of these isozymes in T cells.

### PROLIFERATIVE EFFECTS OF INDIVIDUAL PKC FAMILY MEMBERS

#### PKCα

Use of selective pharmacological inhibitors, antisense technology, or siRNA has identified an anti-proliferative and differentiation-inducing role of PKCα in multiple cell types, e.g., intestinal epithelial cells, keratinocytes, mammary epithelial cells, and melanoma cells (Black, 2000, 2010). Anti-proliferative effects of PKCα affecting G1 → S transit include downregulation of cyclin D1 (Detjen et al., 2000; Hizli et al., 2006; Guan et al., 2007), as well as induction of p21^Cip1^ (Frey et al., 1997, 2000; Abraham et al., 1998; Slosberg et al., 1999; Black, 2000; Detjen et al., 2000; Tibudan et al., 2002; Clark et al., 2004; Matsumoto et al., 2006) and p27^Kip1^ (Frey et al., 1997, 2000; Detjen et al., 2000; Tibudan et al., 2002). Induction of p21^Cip1^ is also involved in the ability of this isozyme to delay S phase transit and induce G2/M arrest (Frey et al., 1997; Oliva et al., 2008). Our analysis in intestinal epithelial cells indicated that downregulation of cyclin D1 represents one of the earliest effects of PKCα signaling (Frey et al., 2004; Hizli et al., 2006): PKCα-induced loss of cyclin D1 results from translational and transcriptional inhibition, mediated by activation of the translational repressor 4E-BP1 and downregulation of the Id family of transcription factors, respectively (Clark et al., 2004; Hizli et al., 2006; Guan et al., 2007; Hao et al., 2011). Suppression of cyclin D1 expression by PKCα can involve different intermediate signaling events, including activation of the ERK/MAPK pathway (Clark et al., 2004; Hizli et al., 2006; Guan et al., 2007; Hao et al., 2011) and RORα-mediated suppression of Wnt/β-catenin signaling (Bird et al., 1998). Consistent with a role of PKCα in growth inhibition, activation/membrane association of this isozyme is detected in post-mitotic cells in the intestinal epithelium (Saxon et al., 1994; Frey et al., 2000) and epidermis (Tibudan et al., 2002) *in vivo*. Furthermore, PKCα knockout mice show increased proliferative activity within intestinal crypts, and the tumor suppressive activity of this isozyme in the intestine has been linked directly to its effects on the cell cycle machinery (Oster and Leitges, 2006; Pysz et al., 2009).

Growth-stimulatory effects of PKCα have been reported in glioma cells, osteoblasts, chick embryo hepatocytes, hepatocellular carcinoma cells, and myoblasts, among others (Black, 2000, 2010). Proliferative effects of PKCα on the cell cycle machinery include increased levels of cyclin D1 and cdk4, and enhanced cyclin/cdk2 complex activity (Zhou et al., 2002; Alisi et al., 2004; Wu et al., 2008; Lovatt and Bijlmakers, 2010). PKCα can also elicit a p21^Cip1^-dependent enhancement of proliferation as seen in glioma cells (Besson and Yong, 2000). The ability of PKCα to promote proliferation has been linked to signaling through the ERK/MAPK pathway (Schonwasser et al., 1998; Shatos et al., 2008).

Consistent with the cell cycle effects of PKCα described above, this isozyme is targeted by various physiological stimuli that elicit changes in proliferation (Bird et al., 1998; Black, 2000, 2010). Interestingly, PKCα can mediate opposing cell cycle-specific effects of these agents depending on context. For example, PKCα appears to mediate both proliferative (Buitrago et al., 2003) and growth-inhibitory (Chen et al., 1999; Bikle et al., 2001) effects of vitamin D in different systems. This dichotomy has even been observed in cells of the same tissue origin: decreased PKCα expression mediates all-*trans* retinoic acid (ATRA)-induced inhibition of G1 → S progression in SKRB-3 breast cancer cells (Nakagawa et al., 2003), whereas PKCα is required for ATRA-induced growth arrest in T-47D breast cancer cells (Cho et al., 1997).

A role for PKCα in positive regulation of proliferation in T cells was suggested by the finding that, unlike wild-type cells, T lymphocytes from transgenic mice overexpressing PKCα were able to proliferate in response to soluble anti-CD3 antibody (Iwamoto et al., 1992). This role was confirmed by studies of PKCα knockout mice: while PKCα was not required for differentiation of CD4^+^ and CD8^+^ cells or activation-induced IL-2 production, PKCα^-^^/^^-^ T cells showed severe defects in TCR-induced proliferation and IFN-γ production (Pfeifhofer et al., 2006). These effects were specific to T cells since B cell proliferation was unaffected (Pfeifhofer et al., 2006; Gruber et al., 2009).

Interestingly, PKCα and PKCθ cooperate in regulation of T cell proliferation: while PKCα^-^^/^^-^ and PKCθ^-^^/^^-^ showed only a mild activation defect in a graft-versus-host model, double PKCα/PKCθ knockout mice had a severe defect in alloreactive T cell proliferation (Gruber et al., 2009). This effect is of direct physiological relevance since the double knockout mice had significantly improved transplant survival compared with single knockout and control animals (Gruber et al., 2009). These studies further indicated that the cooperative effects of PKCα and PKCθ are due to a combinatorial effect on NFAT activation. A role for this pathway in effects of PKCα is also supported by the fact that constitutively active PKCα can activate NFAT (and AP1) in T cells (Genot et al., 1995). While these studies indicate that PKCα and PKCθ have overlapping functions in regulation of the alloimmune response and NFAT activation, these isozymes clearly have non-redundant functions in T cells. PKCα^-^^/^^-^ mice show a defect in Th1-dependent IgG2a/b switching, indicating that PKCα is particularly important in Th1 cells (Pfeifhofer et al., 2006), a role which contrasts with the more prominent function of PKCθ in Th2 function (Salek-Ardakani et al., 2004). These non-redundant actions of PKCα may reflect its recently identified role in phosphorylation of Akt on serine 473 in T cells (Yang et al., 2010). The relevance of this phosphorylation is supported by the finding that Akt links mTORC2 to Th1 cells whereas PKCθ regulates mTORC2-mediated Th2 differentiation (Lee et al., 2010).

#### PKCβ

The two major splice variants of the PKCβ gene (*prkcb*), PKCβI and PKCβII, have different functions; however, the fact that early studies did not always differentiate between these forms, and knockdown and knockout strategies can affect both isoforms, has complicated interpretation of their individual roles.

The cell cycle-specific effects of PKCβII, which have been noted in both G1 and G2/M phases, appear to be largely stimulatory (Black, 2010). Effects in G1 have been ascribed to the ability of PKCβII to enhance transcription of cyclin D1 (Li and Weinstein, 2006), promote pRb phosphorylation (Suzuma et al., 2002), or to stimulate CAK activity through direct phosphorylation (Acevedo-Duncan et al., 2002). Studies by Fields and colleagues have established that phosphorylation of lamins contributes to the effects of PKCβII on G2 → M transition (Goss et al., 1994; Walker et al., 1995; Thompson and Fields, 1996; Murray and Fields, 1998), while studies by Newton and colleagues (Chen et al., 2004) have also determined that PKCβII can affect M phase by regulation of cytokinesis through interaction with pericentrin. However, PKCβII can also inhibit proliferation and induce differentiation in some cell types, with induction of p21^Cip1^ and loss of Cdc25 potentially mediating this activity (Yoshida et al., 2003; Cejas et al., 2005). The PKCβI splice variant has been implicated in positive and negative regulation of proliferation in fibroblasts and colon cancer cells, respectively (Housey et al., 1988; Choi et al., 1990; Sauma et al., 1996); however, these findings relied exclusively on overexpression and further work will be required to determine the specific involvement of the PKCβI isozyme in these effects.

A number of studies indicate that PKCβI and/or PKCβII are involved in regulation of T cell proliferation. For example, antisense-mediated knockdown has implicated PKCβ isozyme(s) in IL-2 signaling (Gomez et al., 1995). Furthermore, PKCβ forms are likely involved in cytoskeletal changes following T cell activation. PKCβII localizes to a cytoskeletal aggregate that forms in close proximity to the microtubule organizing center following T cell activation (Black et al., 1988; Gregorio et al., 1992, 1994) and PKCβI has been shown to associate with microtubules in T cells and to play a role in T cell polarization (Volkov et al., 2001). Since cytoskeletal changes appear to be an important aspect of T cell activation (Repasky and Black, 1996; Martín-Cófreces et al.,2008; freces et al.,2008; ; Alarcónetal.,2011 2011), these observations are likely to be relevant to T cell signaling. This idea is supported by the finding that antisense-mediated knockdown of PKCβI reduced nuclear translocation of NFAT in TCR/CD28-stimulated Jurkat T lymphoma cells (Dreikhausen et al., 2003). However, PKCβ isozymes do not have an essential role in T cell function since PKCβ^-^^/^^-^ mice have no appreciable T cell-related defects. This contrasts with a critical role for PKCβ in B cell receptor signaling (Thuille et al., 2006) and in dendritic cell differentiation (Farren et al., 2010). Thus, any role of PKCβI/II association with cytoskeletal elements is likely to be redundant. In this regard, it is noteworthy that T cell activation leads to translocation of PKCα and PKCθ to the same PKCβII-associated cytoskeletal aggregate described above (J. D. Black and E. A. Repasky, unpublished data; Wang et al., 1999).

#### PKCδ

PKCδ broadly inhibits cell cycle progression in G1 in response to pharmacological agonists and physiological activators such as ATRA, inositol hexaphosphate (IP6), interferons, and testosterone (Watanabe et al., 1992; Fukumoto et al., 1997; Ashton et al., 1999; Uddin et al., 2002; Kambhampati et al., 2003; Nakagawa et al., 2005; Vucenik et al., 2005; Cerda et al., 2006; Bowles et al., 2007). Effects on G1 → S phase progression are mediated by direct or indirect targeting of cyclin D1, cyclin E, cyclin A, p21^Cip1^, and/or p27^Kip1^ (Fukumoto et al., 1997; Vrana et al., 1998; Ashton et al., 1999; Nakagawa et al., 2005; Cerda et al., 2006; Afrasiabi et al., 2008). Cyclin D1 expression is downregulated by PKCδ in colon cancer cells (Cerda et al., 2006; Pysz et al., 2009), as well as in PKCδ overexpressing vascular smooth muscle cells (Fukumoto et al., 1997), primary bovine airway smooth muscle cells (Page et al., 2002), and NIH3T3 cells (Soh and Weinstein, 2003). Consistent with these findings, loss of PKCδ activity resulted in increased levels of cyclin D1 in colon cancer cells (Cerda et al., 2006) and bovine airway smooth muscle cells (Page et al., 2002). PKCδ has also been shown to inhibit mitosis in CHO cells and 3Y1 murine fibroblasts (Watanabe et al., 1992; Kitamura et al., 2003).

Although the majority of studies have detected a growth-inhibitory role for PKCδ, it can also act as a positive regulator of the cell cycle (Kitamura et al., 2003; Cho et al., 2004; Jackson and Foster, 2004; Czifra et al., 2006). PKCδ can enhance G1 → S transit through increased expression of cyclin D1, cyclin E, cyclin A, and/or cdk2 (Kitamura et al., 2003; Santiago-Walker et al., 2005; Grossoni et al., 2007), destabilization of p21^Cip1^ (Santiago-Walker et al., 2005; Walker et al., 2006), reduced nuclear localization of p21^Cip1^ (Sipeki et al., 2002; Ranta et al., 2011), and increased E2F promoter activity (Nakaigawa et al., 1996). In many cases, these effects are mediated by the ERK/MAPK pathway (Jackson and Foster, 2004; Grossoni et al., 2007). The opposing effects of PKCδ on cell cycle progression may be regulated by differential phosphorylation on Tyr155 (Acs et al., 2000; Steinberg, 2004).

T cells from PKCδ knockout mice are hyperproliferative and produce more IL-2 cytokine upon stimulation in response to allogeneic MHC. Thus, consistent with a predominant growth-inhibitory role of PKCδ in other systems, this isozyme appears to negatively regulate T cell proliferation, an effect that has been ascribed to attenuation of TCR/CD3-mediated signaling (Gruber et al., 2005a). A similar negative effect of PKCδ on proliferation is also seen in B cells (Miyamoto et al., 2002).

#### PKCε

PKCε generally mediates pro-proliferative responses, and its effects appear to be predominantly in G1/S rather than G2/M (Graham et al., 2000; Balciunaite and Kazlauskas, 2001). The enzyme has been implicated in mediating PDGF-induced G0/G1 → S progression (Balciunaite and Kazlauskas, 2001). Loss of PKCε activity in NSCLC cells is associated with induction of p21^Cip1^, prolonged G1 → S transition in response to serum, and reduced activation of cdk2 complexes (Bae et al., 2007), indicating that this isozyme suppresses p21^Cip1^ accumulation to facilitate cell cycle progression. PKCε can also induce cyclin D1 transcription and upregulate cyclin D1 and cyclin E protein (Soh and Weinstein, 2003; F. Hao, M. A Pysz, A. R. Black, and J. D. Black, unpublished data). Although PKCε is generally downregulated during differentiation (e.g., Yang et al., 2003), the enzyme promotes adipogenic commitment and is essential for terminal differentiation of 3T3-F442A preadipocytes (Webb et al., 2003). Its expression is also enhanced during myogenic differentiation, resulting in upregulation of cyclin D3 (Gaboardi et al., 2010).

The ability of constitutively active PKCε to activate NFAT and AP1 in Jurkat T lymphoma cells points to a role for this isozyme in T cell activation (Genot et al., 1995). Antisense-mediated knockdown has also implicated this isozyme in IL-2 signaling in T cells (Gomez et al., 1995). Furthermore, siRNA-mediated knockdown of PKCε in CD4^+^ T cells severely reduced proliferation *in vitro* and enhanced the growth-inhibitory effects of transforming growth factor beta (TGF-β; Mirandola et al., 2011). These findings support a predominantly growth-stimulatory role of PKCε in T cells, as seen in other systems (see above). However, PKCε^-^^/^^-^ mice show no defects in T cell differentiation, proliferation or activation, indicating that the functions of this isotype may be in large part redundant, at least in the mouse (Gruber et al., 2005b). In contrast to this finding, analysis of Hashimoto thyroiditis patients points to a potential clinical relevance for proliferative effects of PKCε in T cells. These patients had significantly higher expression of PKCε in their T cells compared with healthy controls (Mirandola et al., 2011). Furthermore, while Hashimoto thyroiditis-derived T cells had diminished TGF-β responses compared with healthy controls, knockdown of PKCε in these cells restored normal responsiveness to TGF-β (Mirandola et al., 2011).

#### PKCη

PKCη has been associated with post-mitotic cells in a number of tissues including squamous epithelia (Kashiwagi et al., 2002; Breitkreutz et al., 2007), the epidermis (Breitkreutz et al., 2007), and the intestinal epithelium (Osada et al., 1993). Consistent with this localization, PKCη upregulated p21^Cip1^ and p27^Kip1^, decreased cdk2 kinase activity, and induced growth arrest in NIH3T3 cells and keratinocytes (Livneh et al., 1996; Ishino et al., 1998; Cabodi et al., 2000). However, this isozyme can also enhance proliferation as seen in MCF-7 breast cancer cells, where it upregulated cyclin D and cyclin E levels and promoted a redistribution of p21^Cip1^ and p27^Kip1^ from cdk2 to cdk4 complexes (Fima et al., 2001).

PKCη is recruited to the immune synapse, pointing to involvement of this isozyme in T cell activation (Fu and Gascoigne, 2012). This role was confirmed by the finding that PKCη^-^^/^^-^ T cells have a defective proliferative response to anti-CD3 stimulation *in vitro* (Fu et al., 2011). A somewhat more severe proliferative defect was also observed in response to antigen presentation both *in vitro* and *in vivo* (Fu et al., 2011). Consistent with a role for PKCη in mediating TCR signaling, activated PKCη^-^^/^^-^ T cells showed a reduction in calcium flux and NF-κB translocation (Fu et al., 2011). While these effects are largely redundant with PKCθ, specific effects of PKCη were seen in T cell homeostatic proliferation, which involves self-antigen recognition and IL-7 and IL-15 signaling (Fu and Gascoigne, 2012). Notably, no defect in homeostatic proliferation was seen in PKCθ^-^^/^^-^ mice, indicating that this effect is largely specific to PKCη, although double knockouts did have a somewhat more severe phenotype.

#### PKCθ

PKCθ has been implicated as a positive regulator of proliferation in a number of cell types including gastrointestinal stromal tumor cells and breast cancer cells, where it represses expression of p21^Cip1^ and/or p27^Kip1^ (Belguise and Sonenshein, 2007; Ou et al., 2008), and in capillary endothelial cells, where it promotes G2/M progression (Tang et al., 1997).

A large body of evidence has emerged to support a critical role for PKCθ in T cell activation. The functions of this isozyme are the subject of several excellent reviews in this issue (e.g., Freeley and Long, 2012; Isakov and Altman, 2012; Wang et al., 2012) and will only be discussed briefly here. While PKCθ is dispensable for differentiation of CD4^+^ and CD8^+^ T cells, it is intimately involved in T cell activation and transduces pro-proliferative signals in multiple pathways, including those triggered by the TCR, CD28, and TNF-α (Altman et al., 2000; So and Croft, 2012). As mentioned above, PKCθ is recruited to the immune synapse early in T cell activation, where it is required for formation of the CBM complex, which plays a central role in mediating downstream signaling during T cell activation (Rawlings et al., 2006). In keeping with this role, PKCθ signaling activates a number of transcription factors that regulate T cell activation and proliferation, including Ap1, NF-κB, and NFAT (Pfeifhofer et al., 2003). Studies using *prkcq* knockout mice have determined that PKCθ plays a central role in mediating proliferative responses during T cell activation. PKCθ-deficient T cells lose the ability to proliferate in response to TCR/CD28 activation *in vitro* (Sun et al., 2000; Pfeifhofer et al., 2003). A role for PKCθ in T cell expansion *in vivo* was also apparent from the defective proliferation seen in PKCθ^-^^/^^-^ mice during allergic asthmatic reactions and in response to bacterial infection (Salek-Ardakani et al., 2004; Sakowicz-Burkiewicz et al., 2008).

As seen with PKC isozymes in other cell types, the action of PKCθ in proliferation appears to be highly context-dependent. For example, while a clear role for this isozyme in regulation of Th2 cell proliferation *in vivo* is seen in the allergic asthmatic response, this was not the case for Th1 cells (Salek-Ardakani et al., 2004). Furthermore, PKCθ-deficiency does not affect T cell proliferation in response to viral infection (Giannoni et al., 2005) and can mediate growth-inhibitory effects of cytokine withdrawal (Li et al., 2006b). Notably, while PKCθ generally plays a positive role in proliferation of effector T cells, it has the opposite effect in Treg cells, where it is sequestered from the immune synapse and promotes growth inhibition (Zanin-Zhorov et al., 2010).

A recent study has given insight into possible explanations for divergent functions of PKCθ (Kong et al., 2011). PKCδ and PKCθ are highly homologous; yet, as noted above, PKCδ is growth inhibitory in T cells. In keeping with these differences, PKCδ is not targeted to the immune synapse, disrupts signalosome assembly and cannot substitute for PKCθ in T cell function. These differences are due to a proline-rich motif in the V3 region of PKCθ that mediates indirect interaction with CD28 through Lck. Mutation of this sequence blocks localization of PKCθ to the immune synapse; conversely, a PKCδ mutant containing this sequence was targeted to the immune synapse and could substitute for PKCθ in T cell signaling (Kong et al., 2011; Isakov and Altman, 2012). These findings point to the importance of alterations in protein–protein interactions and localization in dictating the effects of PKC signaling, and offer a mechanism for the divergent roles of PKC isozymes in different cell types and in different signaling environments.

#### Atypical PKC isozymes

While analysis of the functions of atypical PKCs is less advanced than that of other PKC isotypes, PKCι and PKCζ generally appear to promote cell cycle progression. Consistent with a cell cycle stimulatory role of PKCζ, keratin-induced blockade of HaCaT cell cycle progression involved inhibition of PKCζ activity, a reduction in cyclin D1 and cyclin E levels, and pRb hypophosphorylation (Paramio et al., 2001). PKCζ can mediate transcriptional activation of cyclin D1 downstream of Ras (Kampfer et al., 2001), and can induce phosphorylation and proteasome-dependent degradation of p21^Cip1^ downstream of PI-3K (Scott et al., 2002). The ability of PKCζ to modulate the subcellular distribution of p27^Kip1^ during cell cycle reentry of quiescent MCF7 cells is also downstream of PI-3K (Castoria et al., 2004). PKCζ may also enhance cdc25 activity to promote G2/M transit in A549 lung epithelial cells, an effect associated with changes in cdk2 activity (Lee et al., 2011; Kang et al., 2012). Exciting studies by Murray, Fields and colleagues have recently identified PKCι as an oncogene which is required for the transformed growth of various human cancer cell types (Fields and Regala, 2007; Murray et al., 2011). Consistent with these findings, PKCι is upstream of PKCζ in Ras-related upregulation of cyclin D1 (Kampfer et al., 2001). PKCι also phosphorylates and activates CAK in response to PI-3K signaling in glioma and neuroblastoma cells (Acevedo-Duncan et al., 2002; Pillai et al., 2011; Desai et al., 2012) and may target cyclin E in ovarian cancer (Eder et al., 2005).

In contrast to PKCα and PKCε, constitutively active PKCζ had no effect on AP1 and NFAT in Jurkat cells (Genot et al., 1995). However, work of Gruber et al. (2008) points to a role for atypical PKCs in PKCθ-mediated pro-proliferative signaling in T cells. These studies found that PKCζ physically interacts with PKCθ in a yeast two-hybrid screen and that PKCζ is a substrate for PKCθ. This physical interaction likely occurs *in vivo* since PKCζ and PKCι are constitutively localized in lipid rafts to which PKCθ is recruited following activation of primary T cells and Jurkat cells. Use of dominant negative mutant proteins further implicated the atypical isozymes in NF-κB induction by PKCθ. In keeping with their common localization and structure, it appears that PKCι and PKCζ can substitute for each other in most T cell functions. Nonetheless, PKCζ function appears to be particularly important for activation of Th2 cells (Martin et al., 2005): while PKCζ knockout did not result in proliferative or signaling defects in naïve T cells, it dramatically inhibited activation of Th2 cells. This effect was reflected in disruption of STAT6, NFAT, and NF-κB activation following stimulation with anti-CD3. The dramatic upregulation of PKCζ noted during Th2 cell differentiation may account for the inability of PKCι to compensate for loss of PKCζ in these cells (Martin et al., 2005; Gruber et al., 2008). The physiological relevance of PKCζ signaling in Th2 cells is seen in the impaired allergic asthmatic response in PKCζ^-^^/^^-^ mice (Martin et al., 2005).

#### Summary and discussion

From the above discussion, it is apparent that PKC signaling plays an important role in regulation of cell proliferation in a broad spectrum of cell types including T cells. PKC activation can either promote or inhibit transit through multiple stages of the cell cycle. The precise effect of PKCs on the cell cycle is highly context-dependent, and is influenced by the specific isozyme involved, the timing and duration of PKC activation, the cell type, and the signaling environment to which the cell is exposed; however, some themes are beginning to emerge. With regard to individual PKC family members, accumulating evidence indicates that PKCα can exert context-dependent inhibitory or stimulatory effects. While PKCδ can have positive effects on cell cycle progression, its effects are generally inhibitory. On the other hand, effects of PKCβII, PKCε, and atypical PKCs appear to be mainly pro-proliferative, while those of PKCη are generally inhibitory.

**FIGURE 2:**
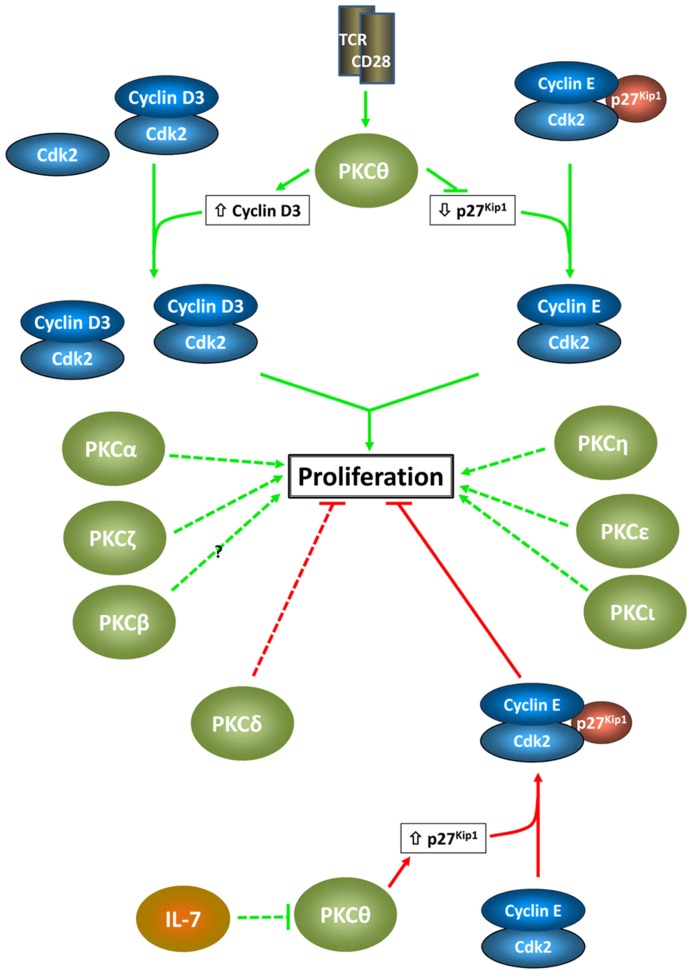
**Proliferative effects of PKC isozymes in T cells**. Positive and negative proliferative effects of individual PKC isozymes are indicated by green arrows and red barred lines, respectively. Proposed cell cycle targets in the growth-inhibitory and growth-stimulatory effects of PKCθ are shown. These targets reflect the importance of D-type cyclins and Cip/Kip ckis as targets for PKC signaling, as seen in other systems (note that in pre-T cells, cyclin D1 appears to be the target for PKCθ). The dashed lines indicate the lack of knowledge of specific cell cycle targets for other PKC isozymes in T cells.

In T cells, multiple PKC isozymes mediate proliferative signals associated with TCR/CD28 engagement (**Figure 2**). These effects, which directly impact immune function, involve both redundant and non-redundant functions of individual PKC family members, and a high degree of cooperation between different PKC isozymes is becoming apparent. As in other systems, the effects of PKC signaling are highly context-dependent, with the reliance on individual isozymes differing between T cell subtypes. While the majority of the characterized effects of PKC signaling in T cells have been pro-proliferative, negative effects are also seen: PKCδ appears to play a predominantly inhibitory role and PKCθ can have negative proliferative effects dependent on the signaling environment and cell type.

Although effects of PKC signaling have been noted in all stages of the cell cycle, the predominant actions of PKC isozymes are in G1 and G2 phases. Similarly, while PKCs can modulate the activity of multiple cell cycle regulatory molecules, consistent with effects in G1 and G2, D-type cyclins and Cip/Kip cdk inhibitors (p21^Cip1^ and p27^Kip1^) are emerging as important targets of PKC control. In keeping with the involvement of these proteins in regulation of quiescence, accumulating evidence indicates that controlling cell cycle entry and exit is an important role for PKC signaling. The ability of PKCs to promote G_0_ → G_1_ progression has been noted in several cell types (Chiu et al., 2002, 2003; Santiago-Walker et al., 2005). PKC signaling has also been shown to promote cell cycle exit in a number of systems, including intestinal epithelial cells, keratinocytes, PKC-overexpressing fibroblasts, and leukemic cell lines (Black, 2000, 2010). Studies in leukemia cells (Zhang and Chellappan, 1996; Vrana et al., 1998; Wang et al., 1998), non-transformed intestinal epithelial cells (Frey et al., 2000), pancreatic cancer cells (Detjen et al., 2000), and keratinocytes (Tibudan et al., 2002) indicate that PKC family members are capable of activating a complete program of cell cycle withdrawal, which can include downregulation of cyclin D1, upregulation of p21^Cip1^ and p27^Kip1^, alterations in the expression and phosphorylation of the pocket proteins p107, pRb, and p130, and changes in E2F expression and complex formation (Zhang and Chellappan, 1996; Saunders et al., 1998). While the ability of PKC signaling to promote exit from quiescence following TCR/CD28 and pre-TCR engagement is established, further studies are required to define its role in promoting cell cycle exit during T cell development and the establishment of quiescent memory T cells.

### SPECIFIC CELL CYCLE TARGETS OF PKC SIGNALING IN T CELLS

Antigen-induced proliferation is a key aspect of both T cell differentiation and clonal expansion (Koch and Radtke, 2011). Thus, the mechanisms underlying PKC isozyme-specific effects on the cell cycle machinery in T cells are of critical importance to immune function. As noted above, the cell cycle is tightly regulated by coordinated actions of cyclins, cdks and ckis, which modulate the activity of the retinoblastoma family and thus expression of E2F-dependent genes (**Figure 1**). Proliferative T cell signaling affects multiple members of this control network. For example, proliferation induced by TCR/CD28 costimulation is associated with increased pRb phosphorylation by cyclin D2/3 and cyclin E, and enhanced transcription of E2F-dependent genes such as cyclins E and A (Colombetti et al., 2006). Analysis of mechanisms underlying these changes has pointed to a particularly important role for cyclin D3, cdk6, and p27^Kip1^ in regulation of T cell proliferation. For example, cyclin D3 and cdk6 knockout mice show defects in T cell proliferation, whereas cdk4 and cdk2 knockout mice do not (Sicinska et al., 2003; Hu et al., 2009), and p27^Kip1^ null T cells show reduced mitogen requirements and are resistant to anergy (Mohapatra et al., 2001; Rowell et al., 2005; Li et al., 2006a).

While PKC activation mediates TCR signaling to NF-κB, NFAT, and Ap1, transcription factors that have been shown to have a direct role in regulation of the cell cycle machinery in T cells, the function of specific PKCs in these effects remains largely unexplored. However, limited information is emerging to indicate that, as in other cell types, D-type cyclins and Cip/Kip proteins are important targets of PKC in these cells. In keeping with the greater attention that has been paid to PKCθ, this evidence primarily concerns the effects of this isozyme. For example, saikosaponins inhibit PKCθ translocation and cause a G0/G1 arrest in activated T cells through downregulation of cdk6 and cyclin D3 and upregulation of p27^Kip1^ protein levels (Leung et al., 2005; Sun et al., 2009). A link to p27^Kip1^ is also supported by the finding that PKCθ loss leads to anergy (Deenick et al., 2010), a process that involves upregulation of this cki (Li et al., 2006a; Wells, 2007, 2009). Through its role in assembly of the CBM signalosome, PKCθ has also been implicated in regulation of cyclin E stability in T cells (Srivastava et al., 2012).

Evidence also points to an ability of PKCθ to regulate cyclin D3 and p27^Kip1^ in pre-T cells. These molecules are downstream of the pre-TCR and PKCθ is an important mediator of signaling from this receptor (Felli et al., 2004; Aifantis et al., 2006; Talora et al., 2006). Pre-TCR activation of PKCθ cooperates with Notch3 to induce cyclin D1 in lymphomagenesis, indicating that this cyclin can also be a target for PKCθ in these cells.

Surprisingly, p27^Kip1^ also appears to be involved in PKC-mediated cell cycle arrest following cytokine withdrawal in T cells. IL-7 withdrawal from the D1 thymocyte cell line results in G1 arrest due to upregulation of p27^Kip1^ (Li et al., 2006b). Notably, PKCθ is activated by IL-7 withdrawal in these cells and the upregulation of p27^Kip1^ could be blocked by a general PKC inhibitor. While these studies do not exclude other PKCs, p27^Kip1^ upregulation was not blocked by the classical PKC inhibitor Gö6976, indicating that the effect was mediated by novel or atypical isozyme(s) (Li et al., 2006b).

### SIGNALING DOWNSTREAM OF PKC IN REGULATION OF THE CELL CYCLE

While cell cycle-specific effects of PKCs can involve direct phosphorylation of cell cycle regulatory molecules (Goss et al., 1994; Acevedo-Duncan et al., 2002; Scott et al., 2002), the effect of PKCs on the cell cycle is generally indirect and involves downstream signaling cascades. Several signaling pathways, including those involving PI-3K/Akt (e.g., Belguise and Sonenshein, 2007; Bakker et al., 2008; Ou et al., 2008) and Wnt-β-catenin (e.g., Gwak et al., 2009; Murray et al., 2009), have been implicated in PKC proliferative signaling. However, analysis of multiple systems has highlighted the Ras/Raf/MEK/Erk pathway as a particularly important mediator of proliferative effects of PKCs. Most members of the PKC family, including PKCα, PKCβ, PKCλ, PKCδ, PKCε, PKCζ, and PKCθ, can target this pathway in many cell types (Kampfer et al., 2001; Chiles, 2004; Clark et al., 2004; Jackson and Foster, 2004; Koike et al., 2006; Bakker et al., 2008). Activation can occur at multiple steps in the Ras–Raf–MEK–Erk cascade. For example, PKCα can intersect the pathway at the level of Ras (Clark et al., 2004) or downstream of Ras through direct phosphorylation of Raf (Kolch et al., 1993). Erk activation mediates the effects of PKC signaling on several cell cycle regulatory molecules, including D-type cyclins and Cip/Kip ckis (Kampfer et al., 2001; Clark et al., 2004; Koike et al., 2006; Matsumoto et al., 2006; Black, 2010; Ranta et al., 2011). Interestingly, Erk signaling can facilitate both positive and negative effects of PKC on cell cycle targets and cell proliferation, and can mediate divergent effects on individual cell cycle molecules even within a single cell type. For example, our analysis has determined that Erk signaling is required for both PKCα-induced cyclin D1 downregulation and PKCε-induced cyclin D1 upregulation in intestinal epithelial cells (Clark et al., 2004; F. Hao, M. A Pysz, A. R. Black and J. D. Black, unpublished data). Thus, in keeping with the complexity associated with the proliferative consequences of PKC activation in general, the effects mediated by Erk signaling are highly context-dependent. While it has been proposed that the duration of activation dictates the proliferative outcome of Erk signaling (cf. Yasuda and Kurosaki, 2008), the anti-proliferative effects of PKCα and the pro-proliferative effects of PKCε both require prolonged Erk activation, with differences in the localization of activated Erk providing a possible explanation for the divergent effects (Clark et al., 2004).

Erk signaling is important for pre-T cell and T cell proliferation (Yasuda and Kurosaki, 2008), pointing to the possible role of a PKC–Erk signaling axis in these cells. It has been proposed that Sos and RasGRP1 cooperate to regulate the sensitivity, duration, and amplitude of Erk signaling in T cells (Yasuda and Kurosaki, 2008). Although analysis of the roles of PKC isozymes in Erk activation in this system is complicated by the fact that RasGRP1 is also a DAG/phorbol ester activated protein (Yasuda and Kurosaki, 2008), siRNA-based analysis has led to the suggestion that PKC may mediate RasGRP1-independent Erk activation in T lymphocytes (Warnecke et al., 2012). This idea opens the possibility that the proliferative response in T cells may be regulated by the coordinated effects of PKC isozymes, Sos-GRB2 and RasGRP1 on Erk activation.

## SUMMARY AND PERSPECTIVES

Although understanding of the impact of PKC signaling on the cell cycle machinery in T cells remains limited, several similarities with other cell types are beginning to emerge (**Figure 2**). As in other cell types, D-type cyclins and Cip/Kip ckis appear to be major targets of PKC signaling in T cells, pointing to effects in G1 and G2. To date, the majority of findings have indicated positive effects of PKCs on cell cycle progression in T cells. However, it should be noted that this may largely reflect a focus on the consequences of T cell activation, which would bias findings in that direction. Evidence for anti-proliferative effects of PKC signaling is indeed accumulating, with PKCδ emerging as a negative regulator. Further analysis is required to identify cell cycle targets which mediate these inhibitory effects. The context-dependence of PKC isozyme-mediated cell cycle regulation observed in other systems has also been noted in T cells, exemplified by the ability of PKCθ to both promote and inhibit T cell proliferation/cell cycle progression. Despite these advances, it is clear that understanding of the cell cycle-specific effects of individual PKC isozymes in T cells is still in its infancy. In addition to delineation of the cell cycle roles of individual PKC isozymes and identification of specific cell cycle targets, issues that remain to be addressed include (a) how the different signaling environments in T cell subsets affect PKC cell cycle signaling, (b) whether PKC signaling plays a role in maintenance of quiescence in T cells and in control of quiescence-related regulators such as FOXO and Krüppel-like transcription factors (Black et al., 2001; Wu and Lingrel, 2004; Vucenik et al., 2005; Hart et al., 2012; Warnecke et al., 2012), and (c) what mechanisms underlie the differential involvement of individual PKCs in T cell proliferation *in vitro* and *in vivo*. Given the emerging importance of mTOR in immune function (Powell et al., 2012), an area of particular interest is the interplay between PKC and mTOR signaling in control of T cell proliferation under the metabolic conditions in which activation occurs *in vivo*. Other areas that remain to be addressed are the relative contribution of direct activation by TCR/CD28 and of activation by secreted cytokines to PKC-mediated proliferative responses, as well as the role of cell survival in the proliferative effects of PKC manipulation, especially *in vivo*. With increasing knowledge of TCR and cytokine signaling and the availability of mouse models for analysis of PKC isozyme function *in vivo*, it is anticipated that a link between PKC and growth-inhibitory signaling in T cells will be confirmed, and that the molecular details underlying the effects of individual PKC isozymes on the cell cycle in T cell subsets will be elucidated in the near future.

## Conflict of Interest Statement

The authors declare that there search was conducted in the absence of any commercial or financial relationships that could be construed as a potential conflict of interest.
